# What to do with chronically sick animals? Pastoralists’ management strategies in the far north region of Cameroon

**DOI:** 10.1186/2041-7136-3-8

**Published:** 2013-03-20

**Authors:** Jessica M Healy Profitós, Mark Moritz, Rebecca B Garabed

**Affiliations:** 1 Department of Anthropology, The Ohio State University, 174 W 18th Avenue, Columbus, OH 43210-1106, USA.; 2 Department of Veterinary Preventive Medicine, The Ohio State University, 174 W 18th Avenue, Columbus, OH 43210-1106, USA.

**Keywords:** Pastoral systems, Brucellosis, Ethnoveterinary research, Livestock breeding strategies, Ecology of infectious diseases

## Abstract

While the goal of African pastoralists is health and longevity of herd and household, some of their management strategies appear to counter this long-term goal. Pastoralists in the far north region of Cameroon, for example, do not always remove chronically sick animals from their herds, even though chronic diseases, such as brucellosis, are contagious and have the potential to cause fertility problems in the herd. We used ethnographic and epidemiologic methods to understand why pastoralists do not remove chronically sick animals and whether their management strategies have an impact on herd fertility. We used semi-structured interviews to collect data on pastoralists’ understandings of disease and its impacts on fertility as well as data on herd management. We compared these data with disease prevalence and herd fertility data to measure the effect of management strategies on herd fertility. We found that the percentage of chronically sick animals in a herd negatively correlated with herd fertility, but this was not true for the prevalence of brucellosis. Thus, preliminary examination of disease costs and benefits suggests that herders’ decisions to keep sick animals in their herds may lower herd fertility, but this is not due to brucellosis alone. The results of this study underline the complexity of infectious disease ecology in pastoral systems and the need for holistic and comprehensive studies of the ecology of infectious diseases in pastoral systems.

## Introduction

The long-term goal of African pastoralists is the health and longevity of their herds (Krätli 2008; Mace 1993; Stenning 1958). Many pastoralist systems are uniquely adapted to a highly unpredictable environment, especially in Africa's arid and semi-arid lands. Pastoralists’ expert knowledge of animal husbandry and of their physical environment allows them to exploit this unpredictability to their advantage (Behnke and Scoones 1993; Krätli and Schareika 2010; Schareika 2003). In his description of the WoDaaBe of Niger, Krätli details how their breeding system is devoted not only to ensuring genetic diversity, but also to ensuring that cattle behaviour necessary for survival, such as selective feeding on the most nutrient-rich grasses, is passed down from generation to generation (Krätli 2008). Health and longevity of the herd is important because herds are passed down from human generation to generation and the survival of the next generation of humans is dependent upon the health of the next generation of the herd (Krätli 2008; Mace 1993; Stenning 1958). But seemingly in direct opposition to this goal, we have found that pastoralists in the far north region of Cameroon do not always remove chronically sick animals from their herds even though these animals may have infectious diseases, like brucellosis, which can cause fertility problems in the herd and illness in humans and animals (John et al. 2010; Scolamacchia et al. 2010).

Bovine brucellosis, one of the most common zoonoses in the world, is highly contagious and can negatively impact herd fertility through abortions, weak offspring, and/or lowered milk production (John et al. 2010; Scolamacchia et al. 2010). Brucellosis bacteria (*Brucella abortus*) can be transmitted from cattle to humans via consumption of raw milk and/or contact with placenta and birth fluids (Acha and Szyfres 2001). Signs in animals are abortions, sterility, inflammation of joints (hygromas), and arthritis (Acha and Szyfres 2001). Acute symptoms in humans are similar to flu and may include fever, sweating, headaches, back pains, and physical weakness, while chronic symptoms are recurrent fever, joint pain, and fatigue (Acha and Szyfres 2001; CDC 2007). Brucellosis is endemic across West Africa although prevalence rates vary significantly across groups of humans and cattle (Chabasse et al. 1985; Domenech et al. 1983; Schelling 2002).

The objective of our ethnographic research project was to describe pastoralists’ understandings of animal disease and its impacts on fertility as well as their management strategies, in order to understand the apparent paradox that pastoralists do not remove chronically sick animals from their herd, even though this may negatively affect the long-term goal of pastoralists. We focused our analysis on brucellosis because this infectious disease is endemic in our study area and a chronic disease that has, potentially, a direct and observable impact on herd fertility.

## Study area and population

The far north region of Cameroon has a semi-arid climate with a single rainy season. During the eight-month dry season, cattle lose considerable weight and become more susceptible to diseases. Whereas sedentary pastoralists rely primarily on feeding cotton seed cakes to cattle so as to overcome the difficulties of the dry season, mobile pastoralists limit weight loss of their animals through transhumance, taking their animals to rangelands with the highest quality and quantity of forage.

The mobile pastoralists in the region belong to different Arab and FulBe groups. The FulBe group consists of Jamaare'en, Mare'en, Alijam'en, Adanko'en, Anagamba'en, and Uuda'en groups. All the groups are highly specialized in animal production. The sedentary pastoralists in our study belong to the FulBe and Musgum groups, but there are also Arab and Tupuri sedentary pastoralists in the region (Moritz et al. 2013).

The veterinary infrastructure is thinly spread over a wide area as there are currently 144 Centres Zootechniques et de Contrôle de Sante Vétérinaire (CSV) in the far north region (>34,000 km^2^). The main functions of the CSV are the annual vaccinations and veterinary health controls at livestock markets and along transhumance and trade routes, not the treatment of livestock diseases. Cattle are vaccinated annually against infectious diseases that cause the greatest losses and have the most readily available vaccines: anthrax, blackleg, lumpy skin disease, haemorrhagic septicaemia, and contagious bovine pleuropneumonia. However, there are many other infectious diseases for which there are no vaccinations available in the far north region, like brucellosis. Previous studies have found that livestock losses attributed by herders to diseases are relatively low (2% to 3%), as most sick animals are sold before they die (Moritz 2003). Our survey data indicate that most self-reported losses are due to *mbooru* (foot-and-mouth disease) and *haahaande* (heart-water), but there are no good biomedical data on what diseases are responsible for livestock losses.

## Methods

This study is part of a larger, interdisciplinary study of the transmission and persistence of infectious diseases in humans and animals in the far north region of Cameroon conducted by the Disease Ecology and Computer Modeling Laboratory at the Ohio State University. The goal of the study presented here was to examine how pastoralists’ understandings of diseases and its impacts on fertility shape their management strategies and what the impact of these strategies is on herd production and reproduction. We conducted semi-structured interviews with 21 pastoralists, whose herds are enrolled in our larger study of the transmission and maintenance of foot-and-mouth disease in the Chad Basin (Ludi et al. Serotype diversity of foot-and-mouth-disease virus circulating in the non-vaccinated population within the Lake Chad Basin of Cameroon, in preparation). The sample consisted of 10 mobile herds and 11 sedentary herds. We only interviewed men because they have the primary responsibility for the care of cattle. There is strict sexual division of labour and sex segregation in Arab and FulBe pastoral households in the far north region of Cameroon; women are responsible for the house, and men are responsible for the herd. Questions and responses were translated from English into Fulfulde (and back) with the assistance of an interpreter, an MA student at the University of Maroua, who had several years of experience working with researchers and herders. All interviews were recorded and transcribed.

First, we asked questions about the calf rope where calves under the age of six months are tethered in the morning and evening to control their access to their mother's milk. During the day, these calves are grazed separately from the main herd, so they only access their mothers at milking times. We documented the health history of 106 calves on the rope as well as the reproductive health history of their mothers, which included information about an additional 222 calves. This provided us with data for a total of 328 calves and 106 cows. The calf rope is a practical tool to measure the fertility of the herd in the past year. We asked questions about the calves’ age, sex, health, as well as the reproductive history of their mothers. Second, we asked about the health of all animals in the herd and the management of diseases. We were specifically interested in animals that were currently and/or chronically sick with *baakaale* (brucellosis) or other reoccurring diseases. Third, we asked about animals that were sold in the previous year and how pastoralists decided which animal to sell. We asked specifically about the sale and removal of chronically sick animals. Finally, we asked about fertility problems, including abortions, and how pastoralists managed these problems.

We discussed diseases and symptoms using Fulfulde terminology and compared pastoralists’ descriptions with those of biomedical diseases to which the FulBe diseases are conventionally translated (Noye 1989; Tourneux 2007). We note that there are no perfect matches between pastoralists’ and western biomedical concepts (see also Grandin and Young 1996; Krönke 2004; McCorkle 1986). The Fulfulde names and conventional biomedical names for the diseases discussed are as follows: *awse*/trypanosomiasis, *baakaale*/brucellosis, *mbooru/njoobu*/foot-and-mouth disease, *haahaande*/heart-water, and *sondaru*/cough (Moritz et al. 2013).

We used grounded theory to analyze the interview data about herders’ conceptual models of diseases and fertility (Charmaz 2006), which meant that interviews were transcribed, read line by line, and coded for themes related to diseases, fertility, animal sales, and chronically sick animals. These codes were then organized into analytical memos that described common themes. The use of grounded theory approach ensured that we could explore themes that were emerging from our data that were not in our original research design.

The health and demographic data from each calf rope allowed us to develop a fertility profile for the herds, including the average age at first calving, the average number of calves born to each cow, number of calves born in the past year per total herd size, and the average number of observed abortions. The survey of 106 mothers whose calves were on the calf rope and their reported reproductive history allowed us to estimate morbidity and fertility patterns in mobile and sedentary herds for a total of 328 calves.

We also had collected biological samples to estimate the seroprevalence of brucellosis in all 21 study herds for two previous years (2010, 2011). The study herds were selected to be representative of different geographical and exposure groups in the far north region (Ludi et al. Serotype diversity of foot-and-mouth-disease virus circulating in the nonvaccinated population within the Lake Chad Basin of Cameroon, in preparation); thus, the herds are not a random population sample but are rather a sample of transhumance orbits representing the range of exposures and management systems in the region. Within each of these herds, five cattle greater than one year of age were sampled and the same animals were sampled each year. Five cattle represented 7% to 33% of the herd, where ‘herd’ is defined as a management unit, i.e. group of animals that graze together every day of the year. Sampling and testing for brucellosis was according to the following procedure, which was approved by the Ohio State University Institutional Animal Care and Use Committee. Cattle were restrained in standing or recumbent position and 10 to 15 mL of blood was collected from the jugular vein into a serum collection tube. The tubes were allowed to stand in the shade for up to four hours until they coagulated. Then the tubes were centrifuged in the field to separate the serum. The serum was transported back to our field laboratory in a cooler and then kept refrigerated for up to two weeks before testing. Batches of serum were tested for *Brucella abortus* reactivity in the field laboratory using the Rose Bengal (Card) test, which has a published sensitivity of 81.2% and a specificity of 86.3% (Gall and Nielsen 2004). The Rose Bengal test was conducted according to the procedure described by OIE (2009) and used as test reagent, and positive and negative controls were purchased from the USDA National Veterinary Services Laboratory, Ames, IA, USA. All samples considered positive in this study showed strong evidence of agglutination, and results were from batches with controls that reacted appropriately.

We used descriptive statistics, chi-squared tests, Mann-Whitney tests, *t* tests, and linear correlations to examine fertility rates and brucellosis prevalence rates of mobile and sedentary herds using GraphPad InStat (version 3.0a) and SPSS (version 19) for Macintosh.

## Results

To answer our two main questions - why do pastoralists keep chronically sick animals in their herd, and does it affect herd fertility - we will first discuss whether pastoralists recognize the infectiousness of chronic diseases and whether they make the link between these diseases and fertility problems. Second, we will discuss pastoralists’ health management practices, including whether and when they remove chronically sick animals. Third, we will discuss whether and how management strategies affect disease prevalence and herd fertility.

## Pastoralists’ understandings of disease and fertility

Pastoralists in the study region considered cattle diseases a major threat to their livelihoods, and they used a variety of strategies to prevent diseases including smoky fires against flies and mosquitoes, prophylactic use of veterinary medicine, and vaccinations. The majority of our informants argued that diseases could cause fertility problems, including abortions. Diseases such as *awse* (trypanosomiasis), *mbooru* (foot-and-mouth disease), *baakaale* (brucellosis) and *haahaande* (heart-water) were mentioned as possible causes of abortions, but biting flies, snakes, and being hit with a stick were also mentioned. While *baakaale* (brucellosis) was frequently mentioned as a disease that can cause abortions in cattle, pastoralists were more concerned with *awse* (trypanosomiasis). Thus, while pastoralists recognized that a range of diseases could potentially cause fertility problems, they were not overly concerned with brucellosis as a cause of fertility problems.

## Treatment and management of chronically sick animals

During the semi-structured interview, herders were also asked to list the number of currently sick animals that they had in their herd as a whole. Most of the pastoralists reported that they had chronically sick animals: 8 of 11 sedentary herds had chronically sick animals with 8% of all sedentary cattle being sick versus 5 of 10 mobile herds with 3% of all mobile cattle being chronically sick. Chronically sick animals were described by herders as animals that required repeated treatment for diseases within a relatively defined time period (e.g. one year) with temporary, but not lasting success. The diseases held responsible for chronic sickness were *awse* (trypanosomiasis; 55% of all chronically sick animals in 21 herds), *mbooru/njoobu* (foot-and-mouth disease; 17%), *sondaru* (cough; 17%), and *baakaale* (brucellosis; 11%). Herders identified the diseases on the basis of symptoms presented; for example, excess saliva and blisters on the mouth and hooves were mentioned as a sign of *mbooru,* while swollen joints were mentioned as a sign of *baakaale.* Only half of all the pastoralists in our study said that they have had animals with *baakaale* (most of them were mobile herders), even though the seroprevalence data showed that all but one herd had animals with antibodies to brucellosis (suggesting infection at some point in time).

All pastoralists used veterinary drugs to treat sick animals, mainly trypanocides and antibiotics, but they used a wait-and-see approach in administering the drugs, which meant that they would administer the first dose of the drug, wait to see if the animal's health improved, and then either stop treatment (if animal improved), administer another dose, or change drugs (if the animal's condition stayed the same or worsened). Traditional treatments were sometimes mentioned, but not by all pastoralists. Thus, pastoralists relied primarily on veterinary drugs to treat animals, which represent an observed monetary cost of having chronically sick animals in a herd. However, the wait-and-see method of using these drugs means that the cost of treatment is relatively small even over long periods of time.

## Removal of chronically sick animals

Thirteen pastoralists said that they had chronically sick animals in their herd, and these animals represented on average about 6% of the animals in the herd. Most of these animals had been sick for half a year, but others had been sick for more than three years. Nevertheless, pastoralists said that they would not sell chronically sick animals, even if the animals were currently not reproducing.

In general, animals were only sold when pastoralists had a pressing economic need for cash. The size of the need determined what animals were sold (see also Moritz 2003). If pastoralists had large needs (e.g. marriage, legal fees), they would sell a large or fat animal. If they had small needs (e.g. food, taxes, medical bills, clothes, wages), they would sell small stock, young male calves, or old cows that were no longer productive. Only if there were no young male calves or old cows would pastoralists sell chronically sick animals.

In general, pastoralists tried to avoid selling sick animals because they fetched low prices. Chronically sick animals were sold under two conditions. First, if death was imminent, pastoralists would either slaughter the animal themselves so that they could consume it or sell the meat, or they would sell the animal immediately on the spot for any price and accept the economic losses. Second, if death was not imminent, pastoral-ists would wait until the animal recovered as much possible so that they could sell the animal at the market and avoid significant economic losses. About 10% of the cattle sold by pastoralists in our sample were reportedly sick at the time of the sale. When asked, ‘Do people buy very sick animals?’ pastoralists explained that there are indeed people who buy sick cattle from time to time because it is a source of' ‘cheap meat’. In fact, it seemed somewhat inappropriate to sell sick animals as some pastoralists argued emphatically that they never sold sick animals, even though the survey data showed that some of them had sold sick animals in the previous year.

While pastoralists in our sample raised animals for subsistence and not for profit, they made economically rational decisions, weighing the costs and benefits of keeping and/or selling chronically sick animals. Because the production costs in agro-pastoral and mobile pastoral systems are relatively low (Moritz 2003), if there was any hope that chronically sick animals would recover, it made economic sense to keep these animals in the herd. The question is whether this decision affected disease prevalence and herd fertility.

## Impact on disease prevalence and herd fertility

**Figure 1:**
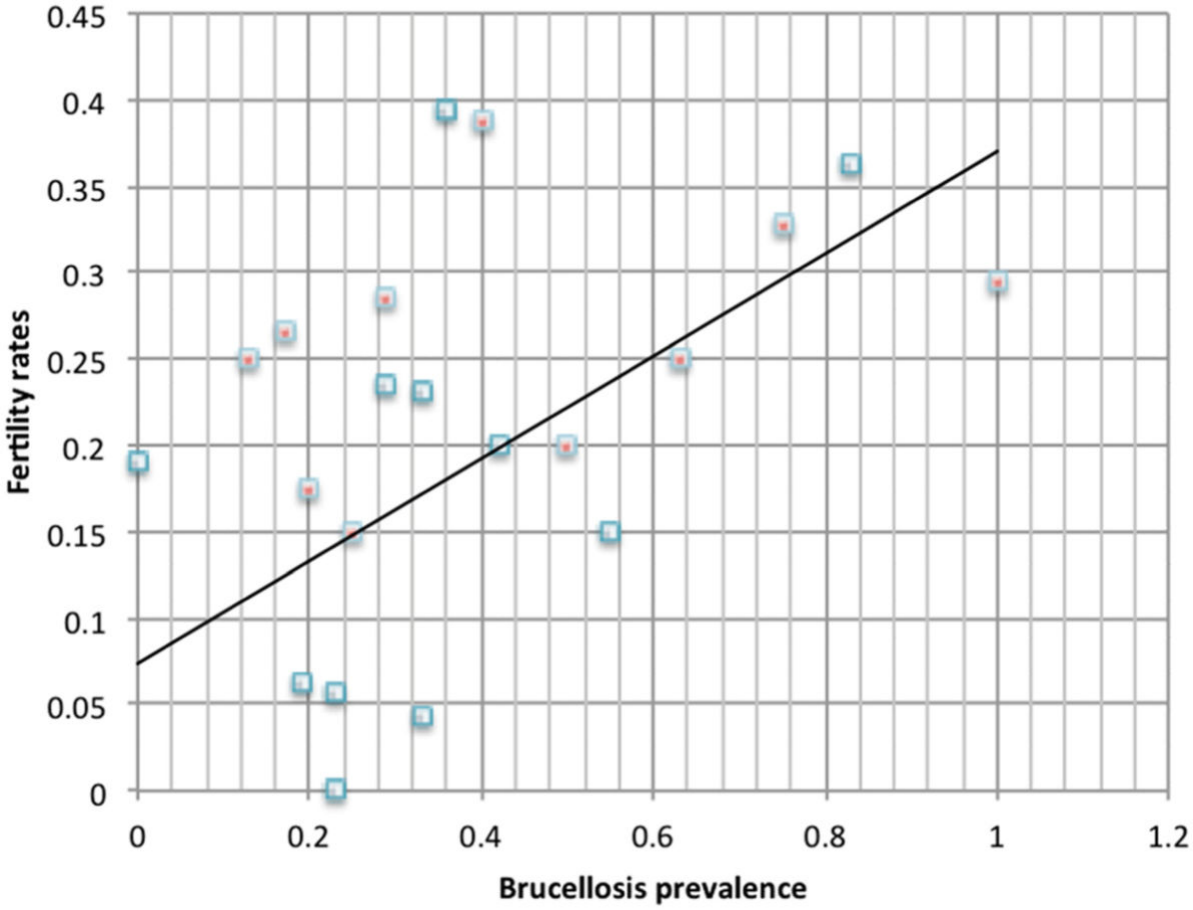
Correlation between brucellosis prevalence and fertility rates in herds There is a statistically significant correlation between brucellosis prevalence and fertility in herds (Pearson's *r* = 0.4385, *p* = 0.0468, *n* = 21. Red speck = mobile herd; blue speck = sedentary herd.)

The seroprevalence data showed that all but one herd had animals infected with brucellosis (Figure 1), with an average of 36% of the animals tested in the herd being infected (Table 1; prevalence of 0.36 adjusted for test accuracy using the Rogan-Gladen estimator, 1978) (95% confidence interval (CI), 0.35:0.38), ranging from a minimum of 0.00 (95% CI, 0.00:0.46) to a maximum of 1.00 (95% CI, 0.54:1.00). We found no statistically significant difference between the prevalence rates of brucellosis in sedentary and mobile herds (see Table 1). While brucellosis is a chronic and endemic disease with potential ramifications for herd fertility, pastoralists were not very concerned about baakaale, and only one of them reported fertility problems caused by *baakaale.*

## Morbidity

About 21% of the calves on the calf rope and about 13% of their mothers had acute health problems, and the main problems were *caarol* (diarrhoea), *awse* (trypanosomiasis), and *mbooru* (foot-and-mouth disease). Other problems for calves were poor appetite, lack of milk, and skin conditions. There were no statistically significant differences between mobile and sedentary herds with regard to reported health problems (morbidity) for calves and their mothers (Table 1).

## Fertility

The average age at which cows in our sample gave birth to their first calf was 4.5 years old. The average age of cows that had a calf on the rope was 7.5 years. The average number of calves that cows in our sample had produced was two with a minimum of one and a maximum of six. The reproductive history of the cows allowed us to estimate a calf mortality rate of 14% (30 of the 222 calves had died). There were no statistically significant differences between mobile and sedentary herds for age at first calving, average age of mothers with current calves, number of calves per cow, or calf mortality (Table 1). Only three observed abortions were reported amongst all the cows surveyed: two among the mobile herds and one among the sedentary herds. The average abortion rate was exactly the same in both groups: 0.03 abortions per cow per year. Finally, herd fertility crudely measured as the number of calves on the calf rope as percentage of the total number of animals in the herd was 13% for sedentary herds and 26% for mobile herds (Table 1). Because the calves on the calf rope are less than six months old, these rates are not the annual fertility rates. All the numbers reported here are within the range observed in other studies in the region (Moritz 2003; Njoya et al. 1997). In short, we found no major differences in morbidity and fertility patterns between mobile and sedentary herds (e.g. age at first calving, birth intervals), except for overall herd fertility, which is higher for mobile herds.

However, we found a surprising positive relationship between brucellosis prevalence and increased herd fertility (Pearson's *r* = 0.4385, *p* = 0.0468, *n* = 21): herds with higher brucellosis rates also had higher fertility rates (see Figure 1). While we realize that our sample is small and we certainly would not suggest that brucellosis actually increases fertility rates, the surprising result might explain why pastoralists were not concerned about *baakaale* (brucellosis) and its impact on herd fertility. It also raises a number of important questions about the study of the ecology of infectious diseases in pastoral systems, which we will discuss in the next section.

However, we did find a negative correlation between the percentage of reported chronically sick animals in the herd and fertility rates (Pearson's *r* = 0.4506, *p* = 0.0404, *n* = 21), but not between the percentage of reported chronically sick animals in the herd and brucellosis prevalence, suggesting that keeping chronically sick animals in the herd lowered overall fertility, but that is likely not due to brucellosis.

## Discussion

In our study, we set out to answer two main questions: (1) why do pastoralists keep chronically sick animals in their herds, and (2) does keeping chronically sick animals in the herd affect herd fertility?

First, pastoralists recognized that a range of diseases could potentially cause fertility problems. They were not overly concerned with *baakaale* (brucellosis); *awse* (trypanosomiasis) was a considered a greater problem. We also found considerable variation in risk perceptions regarding abortion storms (many abortions occurring in a short span of time), and suggestions that pastoralists were more concerned about other infectious diseases like foot-and-mouth disease and contagious bovine pleuropneumonia. Because these herds were all enrolled in a foot-and-mouth disease study and were asked about the disease regularly, there may have been an increased awareness of foot-and-mouth disease in the study population, but this is not expected to have changed their views on any other diseases.

Pastoralists’ first response to sick animals was to use different kinds of veterinary drugs, often in different sequential combinations depending on whether animals recovered or not. We found no qualitative differences in disease management strategies, including the removal of chronically sick animals, between mobile and sedentary pastoralists. Pastoralists avoided selling chronically sick animals because there was always the possibility that animals would recover. Moreover, sick animals (reportedly) fetched considerably lower prices at livestock markets and thus represented an economic loss while the cost of keeping the animal and occasional treatments was seen as relatively low. Only if there was no chance the animal would recover, often after being in the herd for multiple years, would pastoralists sell the animal, waiting for the right moment when the animal could fetch a reasonable price.

We did find a negative correlation between the percentage of chronically sick animals in the herd and herd fertility (keeping in mind that this is based on self-report data from a relatively small sample of herds). Thus, preliminary examination of disease costs and benefits suggests that herders’ decisions to keep sick animals in their herds may lower herd fertility, although it is unclear what the exact mechanism is, i.e. whether it is due to the indirect effects of diseases on animal condition or the direct effects of infectious diseases like brucellosis. For example, although the prevalence of brucellosis was relatively high in our sample (37%), there was no negative correlation between herd-level brucellosis prevalence and herd fertility. On the contrary, we found an unexpected positive correlation.

We are not suggesting that brucellosis has a positive effect on fertility, but these surprising results do have implications for studies of the ecology of infectious diseases in pastoral systems in sub-Saharan Africa. First, in this particular study, we focused on one specific disease (brucellosis) on the assumption that it directly affects herd fertility. However, there are multiple (infectious) diseases that are endemic in the region, and most of them have potentially an effect on fertility, even if the diseases do not directly affect fertility but only indirectly because animals are not in a good condition. This means that in these contexts, researchers should not focus on one disease but instead use a syndemic approach and conduct comprehensive studies of multiple diseases and their interactions. Second, fertility rates are not just affected by the disease burden, but also by nutritional status. In fact, in order to understand the fertility rates in family herds in this pastoral system, one has to use a holistic approach taking into account disease burden of multiple diseases (not just one) as well as the nutritional intake. Presumably, mobile herds have better nutritional intake than sedentary herds because they go on transhumance, and this may explain the higher fertility rates for mobile herds.

McCorkle (1986) argues that it is critical to understand ethnoveterinary systems holistically in order to develop effective policies and animal production strategies that are appropriate and beneficial for both the target animal and human population (see also Zinsstag et al. 2011). Further investigation of this topic would thus need to involve a closer look at the other factors that affect herd fertility beyond disease management strategies and thorough cost-benefit analysis of the effects of keeping sick animals in herds. Studies involving evaluation of animal nutrition, more detailed measures of fertility, immunology, seasonal effects of disease prevalence, and the prevalence of diseases other than brucellosis, in particular trypanosomiasis, need to be conducted in order to tease out the myriad of factors that affect disease transmission and herd fertility in the far north region and to evaluate the order of magnitude that each plays within the system as a whole. In addition, the actual costs of potentially creating more sick animals versus losing money by selling a sick animal would need to be quantified.

In conclusion, our study suggests that herders’ decisions to keep sick animals in their herds may lower herd fertility, but that this is likely not due to brucellosis alone. In our discussion, we have emphasized the complexity of infectious disease ecology in pastoral systems and the need for holistic and comprehensive studies of the ecology of infectious diseases in pastoral systems. 

## Figures and Tables

**Table 1:** Summary statistics for mobile and sedentary herds

Measure	Mobile	Sedentary	Overall average	Unit	95% CI	Statistical test	*p* value
Herd size	58.8	41.3	49.6	Cattle	34.2 to 65.1	Mann-Whitney	0.0362
Chronically sick animals	0.60	2.27	1.48	Cattle	0.46 to 2.49	Mann-Whitney	0.0331
Calf morbidity	21.5	19.0	20.8	Percent	13.0 to 28.5	Chi square	0.9963
Mothers’ morbidity	9.2	19.0	13.2	Percent	6.8 to 19.7	Chi square	0.2194
Mothers’ age at first calving	4.53	4.96	4.5	Years	(3 to 17)^b^	Mann-Whitney	0.2537
Mothers’ average age	7.3	7.88	7.5	Years	(4 to 20)^b^	Mann-Whitney	0.7714
Lifetime calves per mother	2.2	2	2	Calves	(1 to 6)^b^	Mann-Whitney	0.4568
Calf mortality	13.2	14.1	13.5	Percent	9.0 to 18.0	Chi square	0.3286
Observed abortions	0.03	0.03	0.03	Per cow/year	0.01 to 0.05	NA	NA
Fertility rate^a^	26.2	13.4	20.6	Percent	3.3 to 37.9	*t* test, one-tailed^c^	0.0432
Brucellosis Prevalence	46.1	27.4	36.3	Percent	34.9 to 37.7	*t* test, two-tailed	0.4201

a Calves < 6 months old/Total cattle in herd

b range rather than 95% CI

c a one-tailed test was used here because prior work suggested that mobile herds would have higher fertility rates than sedentary herds.
